# Gene network analyses support subfunctionalization hypothesis for duplicated *hsp70* genes in the Antarctic clam

**DOI:** 10.1007/s12192-020-01118-9

**Published:** 2020-05-20

**Authors:** Abigail Ramsøe, Melody S. Clark, Victoria A. Sleight

**Affiliations:** 1 BioArCh, Department of Archaeology, University of York, York, YO1 7EP UK; 2 Department of Earth Sciences, Natural History Museum, London, SW7 5BD UK; 3 British Antarctic Survey, Natural Environment Research Council, High Cross, Madingley Road, Cambridge, CB3 0ET UK; 4 Department of Zoology, University of Cambridge, Downing Street, Cambridge, CB2 3EJ UK; 5 Present Address: School of Biological Sciences, University of Aberdeen, Zoology Building, Tillydrone Avenue, Aberdeen, AB24 2TZ UK

**Keywords:** Heat stress, Hypoxia, Gene duplication, Ribosome, Signalling, Cytoskeleton

## Abstract

A computationally predicted gene regulatory network (GRN), generated from mantle-specific gene expression profiles in the Antarctic clam *Laternula elliptica*, was interrogated to test the regulation and interaction of duplicated inducible *hsp70* paralogues. *hsp70A* and *hsp70B* were identified in the GRN with each paralogue falling into unique submodules that were linked together by a single shared second neighbour. Annotations associated with the clusters in each submodule suggested that *hsp70A* primarily shares regulatory relationships with genes encoding ribosomal proteins, where it may have a role in protecting the ribosome under stress. *hsp70B*, on the other hand, interacted with a suite of genes involved in signalling pathways, including four transcription factors, cellular response to stress and the cytoskeleton. Given the contrasting submodules and associated annotations of the two *hsp70* paralogues, the GRN analysis suggests that each gene is carrying out additional separate functions, as well as being involved in the traditional chaperone heat stress response, and therefore supports the hypothesis that subfunctionalization has occurred after gene duplication. The GRN was specifically produced from experiments investigating biomineralization; however, this study shows the utility of such data for investigating multiple questions concerning gene duplications, interactions and putative functions in a non-model species.

**Electronic supplementary material:**

The online version of this article (10.1007/s12192-020-01118-9) contains supplementary material, which is available to authorized users.

## Introduction

Improvement in the understanding of the genomic content of any non-model species is impeded by the lack of functional annotation. Annotation rates, particularly in environmental invertebrates, are often low, and many genes assembled within an experimentally derived transcriptome are either designated as unknown or require more detailed analysis to extract conservation at the domain level. Annotation can be particularly problematical when attempting to differentiate the putative functions and regulation of duplicated genes. With the decrease in sequencing costs and subsequent increase in amount and quality of RNA-seq data being produced, it is becoming feasible to use sophisticated information-theory analyses, such as ARACNe [(Algorithm for the Reconstruction of Accurate Cellular Networks), which require large datasets of around 100 non-replicated samples (Margolin et al. 2006)], to reverse engineer predicted gene regulatory networks for non-model species. Such networks can provide significant added value to gene annotations and putative functional assignments as they are calculated in an unbiased approach using mutual information obtained from quantitative expression profiles, rather than biased prior gene annotation or database-based predictions. Whilst these types of network calculations have been used to infer in vivo transcriptional regulation in the biomedical literature for some years (Theodoris et al. 2015; Walsh et al. 2017), the approach is only beginning to be applied to non-model organisms well-poised to answer questions of an environmental or evolutionary nature (Antczak et al. 2015). A computationally predicted gene regulatory network (GRN) has recently been produced using ARACNe for the Antarctic clam *Laternula elliptica* (Sleight et al. 2019). This network was produced from mantle-specific shell damage-repair experimental data and, in addition to its contribution to the study of biomineralization, it also provides a resource to investigate questions related to cellular stress responses in *L. elliptica*, for example, the identification of putative differentiated functions of paralogous genes through examination of neighbouring genes.

Previous molecular analyses of the heat shock response of *L. elliptica* had identified a duplication of the inducible form of the 70-kDa heat shock protein [*hsp70A* and *hsp70B*, (Clark et al. 2008)]. Although identified as the inducible form of *hsp70*, both paralogues were constitutively expressed in animals under control conditions, where *hsp70A* expression was similar between tissues and *hsp70B* expression varied in a tissue-specific manner (Clark et al. 2008). The expression of both *hsp70* paralogues was up-regulated in response to acute heat stress and hypoxia (Clark et al. 2008, 2013, 2017). Under each condition, the level of induction was gene- and tissue-specific, with the response to hypoxia revealing additional variation with age (Clark et al. 2013). The retention of duplicated genes within a genome is suggested to occur by the process of subfunctionalization, whereby each of the paralogues evolves additional functions, which are often tissue- or developmental-specific (Force et al. 1999). The production of a GRN for *L. elliptica* provided an opportunity to analyse the predicted regulation of *hsp70* genes and further test the hypothesis that, following duplication, each *hsp70* paralogue was retained due to subfunctionalization, and therefore are contained in separate GRN submodules with different putative functions.

## Methods

Fragments of the duplicated inducible forms of *hsp70* in the Antarctic clam *L. elliptica* were previously identified via degenerate PCR (Clark et al. 2008) and designated *hsp70A* (accession number AM293598.1) and *hsp70B* (accession number AM293600.1). In the present paper, these short fragments were BLAST searched against a recently published in-house mantle transcriptome database for *L. elliptica* (now available at MolluscDB: https://molluscdb.org/ (Caurcel C (2017)) to identify full-length transcripts for further investigation via the computationally predicted GRN (Sleight et al. 2019).

The two *hsp70* paralogues were mapped to network nodes of the GRN that are termed here “clusters” of the GRN, using Cyctoscape (v3.7.1). The term cluster, rather than node, is used due previous methods of data processing. Briefly, 199,321 transcripts generated by a Trinity mantle transcriptome assembly were collapsed into 18,862 expression clusters with shared expression profiles using a self-organizing tree algorithm (SOTA); i.e. transcripts in the same cluster have tightly correlated expression over all the experimental samples in the dataset and were shown to be co-expressed in vivo using mRNA in situ hybridization (Sleight et al. 2019). A brief schematic overview of methods is available in the supplementary material (Supplementary information S1).

Putative divergence of function for each *hsp70* paralogue was investigated via annotations of the transcripts in clusters within a network submodule, where submodules are defined here as the first- and second-neighbour connections to a cluster containing the *hsp* genes of interest. All transcripts in the GRN were previously annotated by Sleight et al. (2019) using tblastx again the NCBI non-redundant dataset (nr). In addition, in the present paper, annotations for each transcript within the *hsp70* first-neighbour submodules were re-annotated via BLAST sequence similarity (tblastx) searching and interrogation of putative functional domains via InterPro (https://www.ebi.ac.uk/interpro/). Finally, the transcripts in the first-neighbour clusters were further BLAST searched (blastx) against the UniProtKB/SwissProt human database (The UniProt Consortium 2019). The resulting first-neighbour human UniProtKB identifiers were entered into STRING-DB, an online server that uses database searches to predict potential protein–protein interactions and enrichment [significance, against a whole human genome background as human annotations were used, was obtained using a statistical overrepresentation Fisher’s exact test, corrected for multiple testing using the Benjamin–Hochberg method to control the false discovery rate (FDR) https://string-db.org/ (Szklarczyk et al. 2017)]. The reconstructed GRN and associated submodules, assembled transcripts, and detailed annotations are all available as supplementary files in Sleight et al. (2019). The raw RNA-Seq data are freely available for download from NCBI SRA (accession PRJNA398984).

## Results

**Fig. 1:**
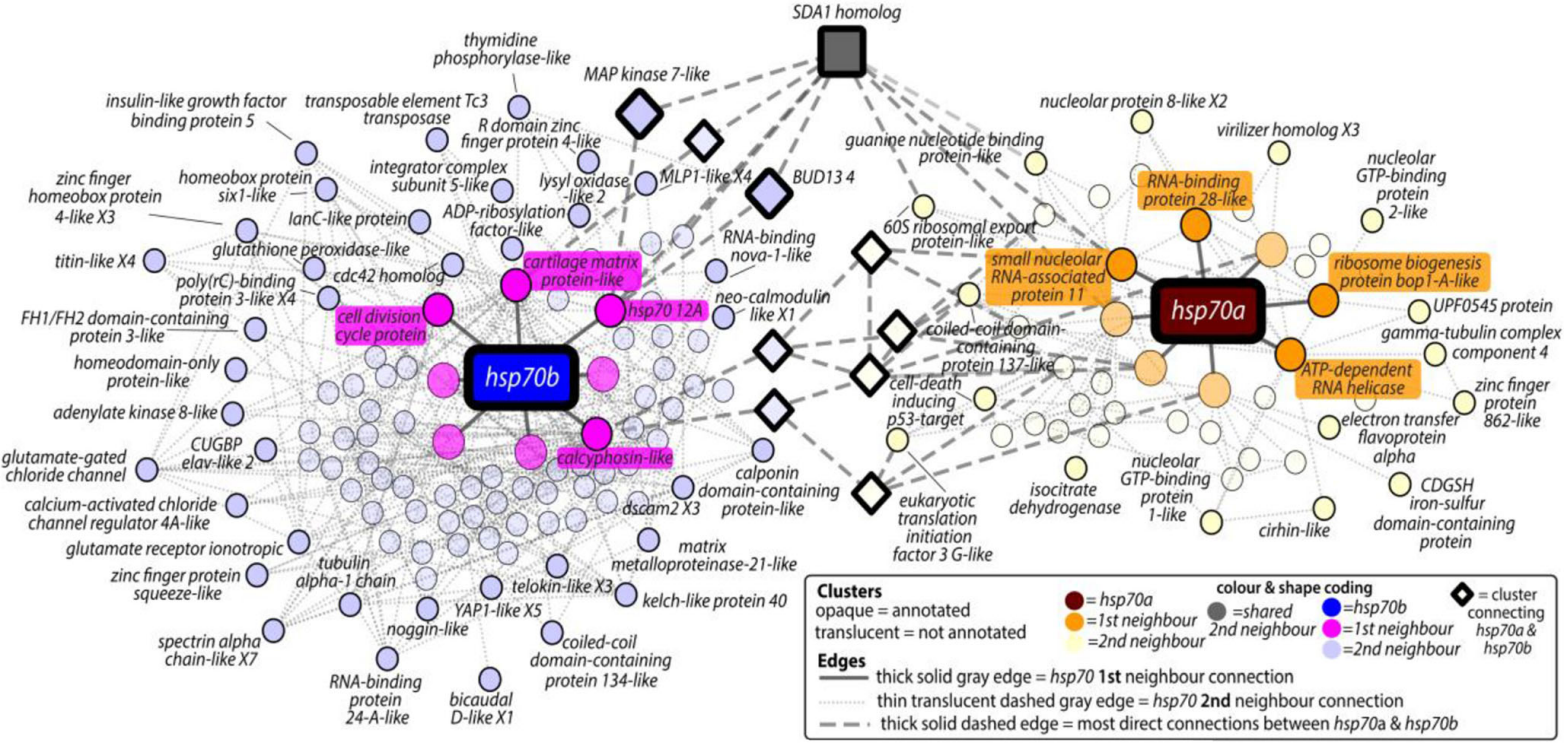
Cyctoscape (v3.7.1) visualization of *hsp70A* and *hsp70B* mapped onto unique submodules of the computationally predicted GRN, linked by a single mutual second-neighbour cluster. Key in bottom right indicates all colour and shape coding for the edges and clusters, which are labelled with a representative transcript NCBI nr annotation

When using the latest *L. elliptica* transcriptome as a database (Sleight et al. 2019), the previously designated *hsp70A* fragment (accession number AM293598.1) matched TRINITY_DN258255_c0_g12 at 99.69% identify, whilst the *hsp70B* fragment (accession number AM293600.1) matched TRINITY_DN246078_c0_g2 at 100% identify. At present only one full-length *hsp70* gene is present for *L. elliptica* in the public databases (GenBank: ABM92345.1) (Park et al. 2007), which matched the Trinity transcript for *hsp70A* (TRINITY_DN258255_c0_g12, probability score = 1293.9, 100% amino acid identify, E value 0.0). When using public database searches (tblastx against nr) the Trinity transcript for *hsp70B* (TRINITY_DN246078_c0_g2) most closely matched the intertidal limpet *Cellana toreuma* HSP70 protein (probability score = 1031.6, 80.6% amino acid identify; 91.8% amino acid similarity, E value 0.0). Comparison of the translated products of these two full-length *hsp70* transcripts in *L. elliptica* revealed 71.7% identity and 84.7% similarity between them at the protein level. Evaluation of these protein sequences showed that both possess all the signature motifs for the HSP70 family (Supplementary information S2). Each *hsp70* paralogue was then mapped onto the GRN, *hsp70A* was located in cluster7829, whilst *hsp70B* mapped to cluster9844 (Fig. 1).

**Fig. 2:**
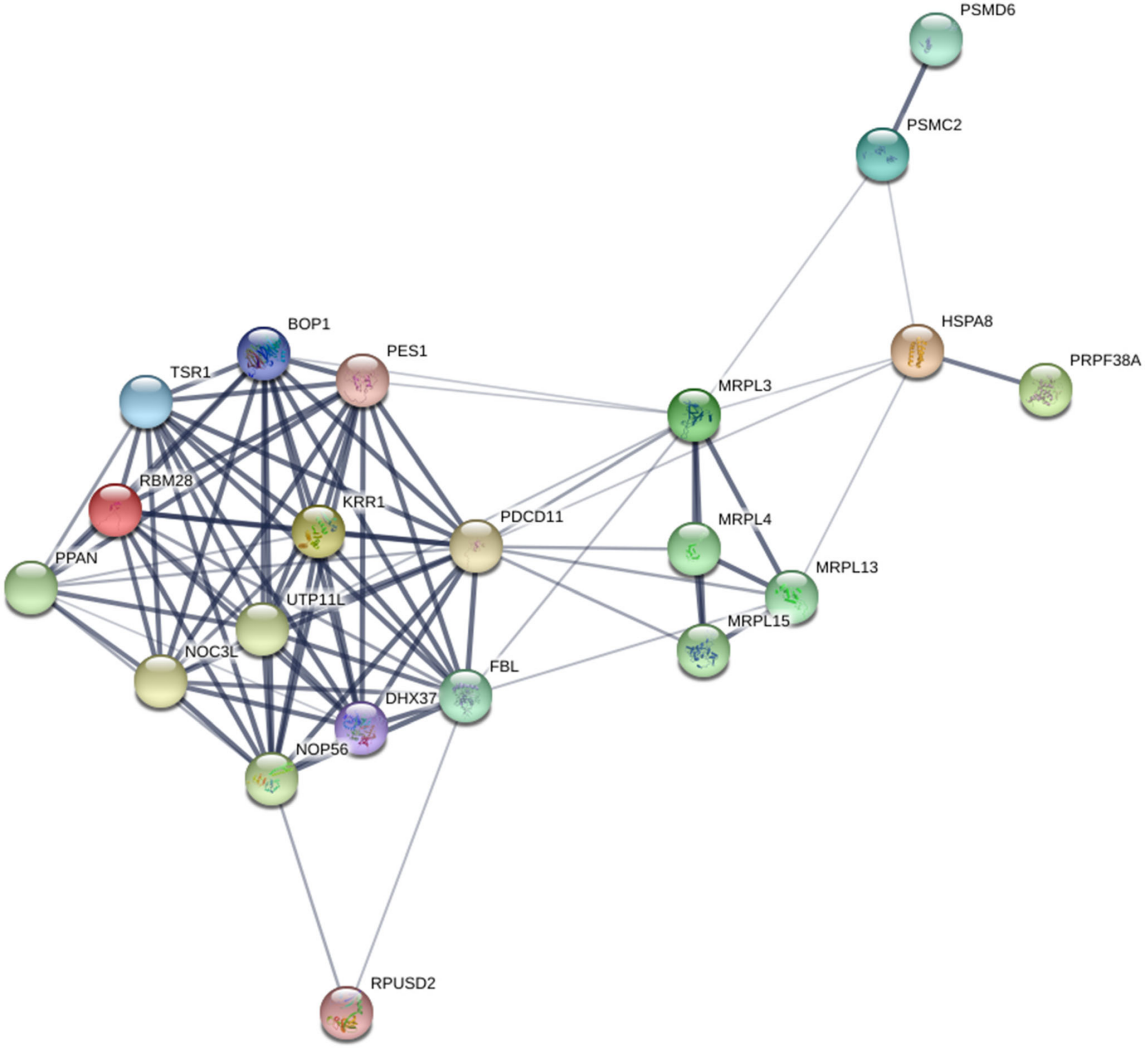
STRING-DB predicted protein–protein interactions for all transcripts within the first-neighbour cluster network of *hsp70A* in the GRN*.* In this diagram, *hsp70A* is represented as HSPA8 (the closest sequence match in the human genome)

*hsp70A*—first neighbours: Cluster7829 containing *hsp70A* includes another transcript, annotated as the hypothetical protein CAPTEDRAFT_177551. Domain annotation of this protein revealed that this was a member of the universal ribosomal protein uL3 family, in the cosmopolitan worm *Capitella teleta*. The *hsp70A* cluster7829 had eight first-neighbour clusters (Fig. 1—orange clusters). Analysis of the annotations associated with the genes in these first-neighbour clusters had functions related to ribosome biogenesis and RNA processing (Supplementary information S3). Database-based STRING-DB analysis of first-neighbour annotations suggested that *hsp70A* directly interacted (with medium confidence or higher) with four of the eighteen genes identified in the first-neighbour clusters (Fig. 2). These comprised MRPL3 (mitochondrial protein L3), PDCD11/RRP5 (essential for generation of mature 18s rRNA), PSMC2 (proteasome component, involved in protein degradation) and PRPF38A (PRP38 pre-mRNA processing factor). *hsp70A*’s first neighbours were functionally enriched in RNA processing, ribosome biogenesis and rRNA processing (Supplementary information S4).

*hsp70B*—first neighbours: Cluster9844 containing *hsp70B* also had eight first-neighbour clusters (Fig. 1—magenta clusters), and the cluster contained a single transcript—*hsp70B*. The first neighbours of cluster9844 did not show a clear functional pattern, and annotation was generally at the domain level (Supplementary information S3). Many annotations were for PDZ domains and two with GPCR (G-protein-coupled receptor). The most notable first-neighbour annotation was contained in cluster7651 with the predicted protein Hsp7012A in *Crassostrea gigas*. STRING-DB analysis of the first-neighbour annotations of *hsp70B* showed no expansive network, with only one protein contained in the first-neighbour clusters identified in the GRN directly interacting with *hsp70B*, namely HSPA12B. There were no other significant interactions, which could have been partly due to low-quality BLAST mapping between the *L. elliptica* transcriptome and the human proteome. The median scores, coverage and median identities between the two *hsp* clusters were notably different: 486, 64%, 55% (7829 and first neighbours) and 129, 21%, 47% (9844 and first neighbours) respectively.

Linkage between *hsp70A* and *hsp70B*—second neighbours: Interrogation of the second-neighbour annotations provided further evidence that *hsp70A* is involved in ribosome biogenesis (Fig. 1—yellow clusters, such as, *nucleolar protein 8-like*, *nucleolar GTP-binding protein 1*, *nucleolar GTP-binding protein 2*). *hsp70B*’s second-neighbour annotation (Fig. 1—purple clusters) provided an additional layer of functional data with many more annotations than the first-neighbour analysis linking to several signalling pathways (such as *MAP kinase kinase 7-like*, *insulin-like growth factor binding protein 5*, *noggin-like*) and transcription factors (such as *homeobox protein six1-like* and *zinc finger protein squeeze-like*), in addition to response to cellular stress (such as *glutathione peroxidase-like* and *cdc42 homolog*) and cytoskeletal trafficking and remodelling (such as *protein bicaudal D-like* and *tubulin alpha-1 chain*). Although in separate submodules, the *hsp70* paralogues were linked via a mutual second neighbour within the GRN (Fig. 1—single grey cluster). Annotation of the genes linking the second-neighbour networks of each paralogue (Fig. 1—diamond/square clusters) was related to protein and nucleic acid binding, whilst some evidence of involvement in ribosome biogenesis was also seen, namely in clusters8788 and 7959. The latter of which being the key mutual second neighbour linking the two first-neighbour networks, and contained an SDA1 homolog, which is required for the export of 60S pre-ribosomal subunits (Supplementary information S4).

## Discussion

Interrogation of the GRN clearly identified *hsp70A* and *hsp70B* in different clusters (7829 and 9844 respectively), with discrete first neighbours. Analysis of the annotations associated with genes within first- and second-neighbour submodules revealed differences in the putative gene regulation and interaction of each *hsp70* paralogue. In particular, *hsp70A* was strongly associated with genes related to ribosome biogenesis and RNA processing. These data were supported by the STRING-DB and enrichment analyses and agree with Truebano et al. (2010), who reported significant changes in expression of ribosomal proteins in *L. elliptica* under heat stress. Other heat stress studies in the goby *Gillichthys mirabilis* and the Antarctic fish *Trematomus bernacchii* reported the same significant variation in genes associated with ribosome biogenesis and protein synthesis (Buckley et al. 2006; Buckley and Somero 2009). Whilst up-regulation of transcriptional machinery, including ribosomal proteins, is often associated with the stress response, another reason for this is thought to be that ribosomal proteins have a stabilizing role in the ribosome (e.g. Beck and De Maio 1994; Cornivelli et al. 2003). Any repression or expression due to heat stress is an effort to protect ribosomal structure and function by replacing damaged regions. Indeed, previous experiments on *L. elliptica* have shown a strong reaction of *hsp70A* not only to heat stress but also to hypoxia and ocean acidification (Park et al. 2007; Clark et al. 2008, 2013, 2017; Cummings et al. 2011).

In contrast, the analysis of *hsp70B* provided less defined first-neighbour interactions. Annotation rates were poor for the first-neighbour clusters and were often at the domain level rather than putative orthologous genes in other species. The most numerous domain identified was PDZ (also known as Discs-large homologous regions), which is often found in diverse membrane-associated proteins with signalling functions (Ponting et al. 1997). Two other annotations within these clusters were to GPCRs, which are membrane-associated receptors involved in signal transduction. The first-neighbour cluster annotations included *hsp70 12A* in *Crassostrea gigas*. This is an atypical *hsp70* gene family, which in vertebrates is not up-regulated in response to stress (e.g. Han et al. 2003); it does, however, appear to have been recruited to the stress response in bivalves. In particular, this gene has been subject to massive duplication in the oyster (*Crassostrea*) lineage and is up-regulated in response to several stresses, including heat and xenobiotics (Zhang et al. 2012; Luchmann et al. 2015). The second-neighbour connections for *hsp70B* also showed strong evidence for cellular stress responses, with many genes being involved in signalling, for example the MAPK-signalling, which has previously been shown to interact with Hsp70 during muscle regeneration (Fan et al. 2018). In addition, an oxidative stress response gene, *glutathione peroxidase-like*, was one of *hsp70B*’s second neighbours, as well as many genes associated with cytoskeletal trafficking and remodelling. It has long been thought that molecular chaperones such as *hsp70* recognize cytoskeletal elements and can both modulate assembly and provide protection (Liang and MacRae 1997). Given the large number of cytoskeletal genes we found in the GRN submodule of *hsp70B*, it is likely that *hsp70B* is playing a role in protecting elements of the cytoskeleton in *L. elliptica*.

The two *hsp70* paralogues were linked in the GRN by a mutual second neighbour, which also contained transcripts involved in ribosome biogenesis and MAPK-signalling. Combining these GRN data with known molecular responses we find that both paralogues are significantly involved in the *L. elliptica* stress response, and in addition, it is seems likely that subfunctionalization has partitioned their potential secondary functions towards ribosome protection (*hsp70A*) and signalling and cytoskeleton protection (*hsp70B*). Hence, despite the known caveats of using primarily in silico methods, the information from the GRN provides valuable clues as to potential additional functions of these inducible *hsp70*s in *L. elliptica*, which can be targeted, and tested more rigorously, in future experiments.

## Electronic supplementary material

ESM 1 (DOCX 513 kb)

ESM 2 (DOCX 17 kb)

ESM 3 (XLSX 44 kb)

ESM 4 (DOCX 12 kb)
