# Supplementary material for: Gene network analyses support subfunctionalization hypothesis for duplicated hsp70 genes in the Antarctic clam
Source: Cell Stress Chaperones. 2020 May 20;25(6):1111–6. doi: 10.1007/s12192-020-01118-9 (PMC7591643; doi:10.1007/s12192-020-01118-9)
Supplement: Supplementary file 1 — (DOCX 513 kb) [file 12192_2020_1118_MOESM1_ESM.docx]

**Supplementary information S1: Schematic overview of the previous methodology used to produce the GRN, and the additional *hsp* analysis in the present paper.**


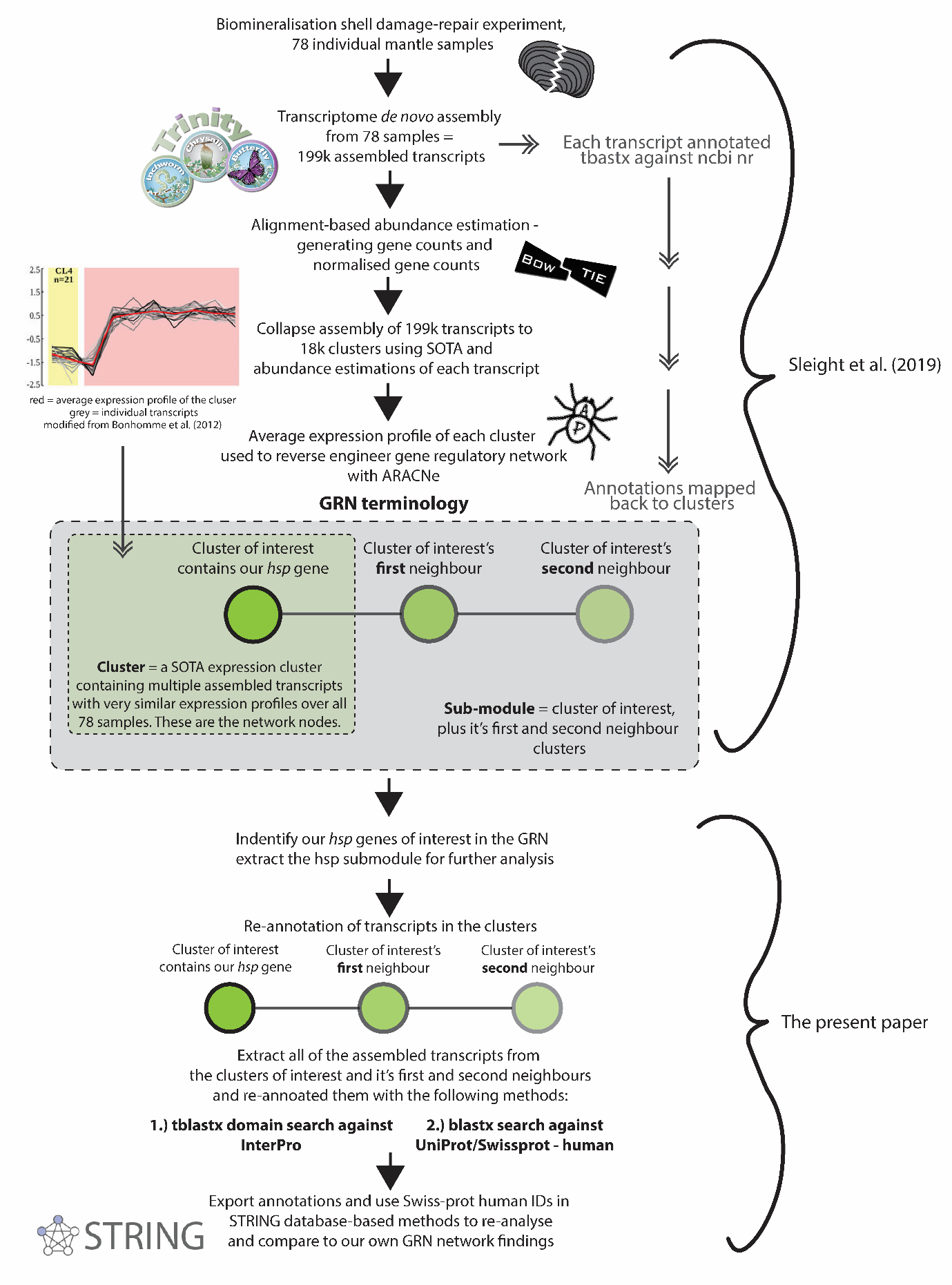


**References:**

Bonhomme, L., Valot, B., Tardieu, F. & Zivy, M. (2012) Phosphoproteome dynamics upon changes in plant water status reveal early events associated with rapid growth adjustment in maize leaves. *Mol Cell Proteomics*, 11(10), 957-72.

Sleight, V. A., Antczak, P., Falciani, F. & Clark, M. S. (2020) Computationally predicted gene regulatory networks in molluscan biomineralization identify extracellular matrix production and ion transportation pathways. *Bioinformatics*, 36(5), 1326-1332.
