# Supplementary material for: Gene network analyses support subfunctionalization hypothesis for duplicated hsp70 genes in the Antarctic clam
Source: Cell Stress Chaperones. 2020 May 20;25(6):1111–6. doi: 10.1007/s12192-020-01118-9 (PMC7591643; doi:10.1007/s12192-020-01118-9)
Supplement: Supplementary file 2 — (DOCX 17 kb) [file 12192_2020_1118_MOESM2_ESM.docx]

**Supplementary information S1: Alignment and annotation of the duplicated inducible *hsp70* transcripts.** Accession number AM293598.1 (*hsp70A*) matched TRINITY_DN258255_c0_g12 at 99.69% identify (abbreviated to TRINITY_DN_258 below). Accession number AM293600.1 (*hsp70B*) matched TRINITY_DN246078_c0_g2 at 100% identify (abbreviated to TRINITY_DN_246 below). These are annotated with the following characteristic signature motifs as described in Rensing & Maier (1994): I: [IVL]-D-[LF]-G-T-T-x-S’ II: D-[LF]-G(3)-T-F-D, III: [TS]-[VC]-P-A-[YN]-[FY]-N, IV: [NP]-[EG]-P-[TS]-A-A and V: R-A-[RK]-F-E-[ED]-[LM]. Also annotated: A = the hydrophobic linker site between the nuclear binding domain (NBD) and the substrate binding domain (SBD) (Zuiderweg *et al.,* 2013) and putative nuclear localisation signal domain (KRK_**_.KDL_***_.RAL_*_R) is denoted with NLS (Rensing & Maier, 1994). In bold: two N-linked glycosylation sites (NKSI and NVSA (consensus of N-X-S)), EEVD which is the cytoplasmic localisation signal and tetra- (GGMP) and penta-peptide (GAGGP) repeats in the C-terminus (Munro & Pelham, 1986; Laursen *et al.,* 1997; Boutet *et al.,* 2003).

**I**

TRINITY_DN258 1 MAKAPAIGIDLGTTYSCVGVFQHGKVEIIANDQGNRTTPSYVAFTDTERL 50

..|||||||||||||||||||||||:|||||||||||||:|||||||

TRINITY_DN246 1 ---MVAIGIDLGTTYSCVGVFQHGKVEIVANDQGNRTTPSYVSFTDTERL 47

TRINITY_DN258 51 IGDAAKNQVAMNPSNTIFDAKRLIGRKFEEQNVQSDMKFWPFTVLSDGGK 100

||||||||.|:||.||:|||||||||:|::..||:|||.|||.:::.|||

TRINITY_DN246 48 IGDAAKNQAAINPENTVFDAKRLIGRRFDDTTVQADMKHWPFKLVNHGGK 97

**III**

TRINITY_DN258 101 PKMQVDYKGEKKTFFPEEISSMVLNKMKDTAEAYLGKTVTNAVVTVPAYF 150

||:|.|||.|.|||.|||||||||.||::|||||||:.|.:|||||||||

TRINITY_DN246 98 PKIQADYKNEMKTFAPEEISSMVLTKMRETAEAYLGQRVKDAVVTVPAYF 147

**IV**

TRINITY_DN258 151 NDSQRQATKDAGTISGLNILRIINEPTAAAIAYGLDKKVGGERNVLIFDL 200

||||||||||||.|:|:|::||||||||||:||||||.:.||:|||||||

TRINITY_DN246 148 NDSQRQATKDAGAIAGINVMRIINEPTAAALAYGLDKNLSGEKNVLIFDL 197

**II**

TRINITY_DN258 201 GGGTFDVSVLTIEDG-IFEVKSTSGDTHLGGEDFDNRMVNHFIQEFKRKH 249

||||||||:|||::| ||||.||:|||||||||||:||||:||:|||||:

TRINITY_DN246 198 GGGTFDVSILTIDEGSIFEVLSTAGDTHLGGEDFDSRMVNYFIEEFKRKY 247

**NLS**

TRINITY_DN258 250 KKDISENKRAVRRLRTACERAKRTLSSSTQASIEIDSLFEGIDYYTSITR 299

|||||:|.|.:||||||||||||.||||::|||||||||||||:|:.|:|

TRINITY_DN246 248 KKDISKNNRTLRRLRTACERAKRALSSSSEASIEIDSLFEGIDFYSKISR 297

**V**

TRINITY_DN258 300 ARFEELNADLFRGTLEPVEKSLRDAKMDKGTINDIVLVGGSTRIPKIQKL 349

||||||.:||||.|:|||||:|||||:||..|:|:|||||||||||:|||

TRINITY_DN246 298 ARFEELCSDLFRSTMEPVEKALRDAKLDKSKIHDVVLVGGSTRIPKVQKL 347

**A**

TRINITY_DN258 350 LQDFFNGKEL**NKS**INPDEAVAYGAAVQAAILHGDKSEEVQDLLLLDVTPL 399

||||.:|||||||||||||||||||||||||.||.|:.::|:||:||.||

TRINITY_DN246 348 LQDFMSGKEL**NKS**INPDEAVAYGAAVQAAILSGDSSDAIKDVLLVDVAPL 397

TRINITY_DN258 400 SLGIETAGGVMTSLIKRNTTIPTKQTQTFTTYSDNQPGVLIQVYEGERAM 449

||||||||||||:|:.|||.||||.:.||||||||||||.|||:||||||

TRINITY_DN246 398 SLGIETAGGVMTTLVARNTKIPTKASNTFTTYSDNQPGVGIQVFEGERAM 447

TRINITY_DN258 450 TKDNNLLGKFELTGIPPAPRGVPQIEVTFDIDANGIL**NVSA**ADKSTGKEN 499

||||||||||||. |||||||||||:|||:||||||:||||.||||||.|

TRINITY_DN246 448 TKDNNLLGKFELV-IPPAPRGVPQIDVTFEIDANGIM**NVSA**QDKSTGKTN 496

TRINITY_DN258 500 KITITNDKGRLSKDDIDRMVNDAEKYKNEDEKQKNRIQAKNSLESYSFNM 549

|||||||||||||::||||||||||:|:||.||:.||.|:|.||||.|::

TRINITY_DN246 497 KITITNDKGRLSKEEIDRMVNDAEKFKDEDAKQRERIAARNQLESYVFSV 546

TRINITY_DN258 550 KSTVEDEKLKDKISEEDKKIILDKCNDVITWLDANQLAETEEFEQQQKDL 599

|..|:.. .||:|:.||:.....|.:.:.|||.|.|||.|||:.:.:::

TRINITY_DN246 547 KQAVDGN--NDKLSDTDKESAQHVCEETLKWLDNNALAEKEEFQHKMEEV 594

TRINITY_DN258 600 EKACNPIVTKLYG**GAGGPGGMPGGMP**GGFGAGAPGEGAAPGGGSGGGPTI 649

:|.|:|::||::||..... |..|.:.|.|...|||:

TRINITY_DN246 595 QKICSPLMTKMHGGEPQQD--------------PDFGQSA**GAGGP**SGPTV 630

TRINITY_DN258 650 **EEVD**  653

||||

TRINITY_DN246 631 **EEVD** 634

**References:**

Boutet I, Tanguy A, Rousseau S, Auffret M, Moraga (2003) Molecular identification and expression of the heat shock cognate (*hsc70*) heat shock protein (*hsp70*) genes in the Pacific oyster *Crassostrea gigas. Cell Stress and Chaperones* **8,** 76-85.

Laursen JR, di Liu H, Wu X-J, Yoshino TP (1997) Heat-shock response in a molluscan cell line: Characterisation of the response and cloning of an inducible HSP70 cDNA. *Journal of Invertebrate Pathology* **70,** 226-33.

Munro S & Pelham HRB (1986) An Hsp70-like protein in the ER: Identity with the 78 kd glucose-regulated protein and immunoglobulin heavy chain binding protein. *Cell* **46,** 291-300.

Rensing SA & Maier U-G (1994) Phylogenetic analysis of the stress-70 protein family. Journal of Molecular Evolution. 38, 80-86.

Zuiderweg ERP, Bertelsen EB, Rousaki A, Mayer MP, Gestwicki JE, Ahmad A (2013) Allostery in the Hsp70 chaperone proteins. *Topics in Current Chemistry* **328,** 99-153.
