# Supplementary material for: Gene network analyses support subfunctionalization hypothesis for duplicated hsp70 genes in the Antarctic clam
Source: Cell Stress Chaperones. 2020 May 20;25(6):1111–6. doi: 10.1007/s12192-020-01118-9 (PMC7591643; doi:10.1007/s12192-020-01118-9)
Supplement: Supplementary file 4 — (DOCX 12 kb) [file 12192_2020_1118_MOESM4_ESM.docx]

**Supplementary Table S1: String-DB enrichment analysis against a whole genome background for *hsp70A* first neighbour clusters**

| **Pathway ID** | **Pathway description** | **False discovery rate** |
| --- | --- | --- |
| **Biological Process** | | |
| GO:0006396 | RNA processing | 1.1e^-05^ |
| GO:0042254 | Ribosome biogenesis | 1.1e^-05^ |
| GO:0006364 | rRNA processing | 2.84e^-05^ |
| GO:0034641 | Cellular nitrogen compound metabolic process | 2.84e^-05^ |
| GO:0010467 | Gene expression | 3.54e^-05^ |
|  | | |
| **Molecular Function** | | |
| GO:0044822 | Poly(A) RNA binding | 1.7e^-18^ |
| GO:1901363 | Heterocyclic compound binding | 3.37e^-06^ |
| GO:0097159 | Organic cyclic compound binding | 3.44e^-06^ |
| GO:0005488 | Binding | 0.00275 |
| GO:0003735 | Constituent of ribosome | 0.00755 |
